# Minimization of metabolic cost of transport predicts changes in gait mechanics over a range of ankle-foot orthosis stiffnesses in individuals with bilateral plantar flexor weakness

**DOI:** 10.3389/fbioe.2024.1369507

**Published:** 2024-05-23

**Authors:** Bernadett Kiss, Niels F. J. Waterval, Marjolein M. van der Krogt, Merel A. Brehm, Thomas Geijtenbeek, Jaap Harlaar, Ajay Seth

**Affiliations:** ^1^ Department of Biomechanical Engineering, Delft University of Technology, Delft, Netherlands; ^2^ Amsterdam UMC Location University of Amsterdam, Rehabilitation Medicine, Amsterdam, Netherlands; ^3^ Amsterdam UMC Location Vrije Universiteit Amsterdam, Rehabilitation Medicine, Amsterdam, Netherlands; ^4^ Amsterdam Movement Sciences, Rehabilitation and Development, Amsterdam, Netherlands; ^5^ Department of Orthopaedics, Erasmus Medical Center, Rotterdam, Netherlands

**Keywords:** ankle-foot orthosis (AFO), plantar flexor weakness, musculoskeletal simulation, predictive simulation, opensim, scone, CMA-ES, reflex-based neuromuscular controller

## Abstract

Neuromuscular disorders often lead to ankle plantar flexor muscle weakness, which impairs ankle push-off power and forward propulsion during gait. To improve walking speed and reduce metabolic cost of transport (mCoT), patients with plantar flexor weakness are provided dorsal-leaf spring ankle-foot orthoses (AFOs). It is widely believed that mCoT during gait depends on the AFO stiffness and an optimal AFO stiffness that minimizes mCoT exists. The biomechanics behind why and how an optimal stiffness exists and benefits individuals with plantar flexor weakness are not well understood. We hypothesized that the AFO would reduce the required support moment and, hence, metabolic cost contributions of the ankle plantar flexor and knee extensor muscles during stance, and reduce hip flexor metabolic cost to initiate swing. To test these hypotheses, we generated neuromusculoskeletal simulations to represent gait of an individual with bilateral plantar flexor weakness wearing an AFO with varying stiffness. Predictions were based on the objective of minimizing mCoT, loading rates at impact and head accelerations at each stiffness level, and the motor patterns were determined via dynamic optimization. The predictive gait simulation results were compared to experimental data from subjects with bilateral plantar flexor weakness walking with varying AFO stiffness. Our simulations demonstrated that reductions in mCoT with increasing stiffness were attributed to reductions in quadriceps metabolic cost during midstance. Increases in mCoT above optimum stiffness were attributed to the increasing metabolic cost of both hip flexor and hamstrings muscles. The insights gained from our predictive gait simulations could inform clinicians on the prescription of personalized AFOs. With further model individualization, simulations based on mCoT minimization may sufficiently predict adaptations to an AFO in individuals with plantar flexor weakness.

## 1 Introduction

The plantar flexor muscles, consisting of soleus and the gastrocnemius, are often weakened in persons with neuromuscular disorders, such as Charcot-Marie-Tooth disease and poliomyelitis (Neumann, 2004; Rossor et al., 2012). Weakness of the plantar flexors results in an altered gait pattern, characterized by reduced push-off power, and excessive ankle dorsiflexion and knee flexion during stance (Steele et al., 2015; Ploeger et al., 2017). These gait deviations lead to a lower walking speed (Waterval et al., 2018) and an elevated metabolic cost of transport (mCoT) (Brehm et al., 2006), which limits daily physical mobility (Nollet et al., 1999). Dorsal leaf spring (DLS) ankle-foot orthoses (AFOs) are often prescribed to provide mechanical support during stance, and augment ankle power during push-off which can reduce mCoT. In a DLS-AFO, a leaf spring connects a footplate to a calf casing posterior of the ankle and passively restricts ankle dorsiflexion by producing an external plantarflexion moment when the ankle is dorsiflexed. As a spring, the AFO can store energy when moving into dorsiflexion and release this energy as the ankle moves towards plantarflexion, thereby providing additional positive work during push-off (Hegarty et al., 2017).

In individuals with plantar flexor weakness, the effects of an AFO on improving gait kinematics and kinetics and reducing mCoT have been shown to depend on the stiffness of the leaf spring (Sreenivasa et al., 2017; Ploeger et al., 2019; Waterval et al., 2019). Beginning at low and with increasing AFO stiffnesses, the mCoT first decreases, before increasing at higher stiffness levels, demonstrating a convex relation between AFO stiffness and mCoT with an optimum stiffness where mCoT is minimal (Ploeger et al., 2019; Waterval et al., 2019). As demonstrated in healthy individuals (Alexander, 1989; Bertram and Ruina, 2001; Selinger et al., 2015), minimizing mCoT is prioritized during gait. As such, it can be expected that patients with gait disorders prefer walking with the stiffness that minimizes their metabolic energy cost (Waters and Mulroy, 1999). That people with gait impairments select their gait pattern, at least partly, based on energy cost minimization is supported by findings of Roemmich et al. who showed that people post-stroke change their gait features in a novel treadmill environment to save energy cost (Roemmich et al., 2019). Additionally, Waterval et al. demonstrated that optimizing AFO stiffness towards minimizing energy cost improves treatment outcomes like fatigue and physical functioning compared to AFOs provided in regular care, indicating that minimization of energy cost with AFOs has clinical value (Waterval et al., 2020). In case of plantar flexor weakness, the initial reduction in mCoT is thought to be the result of normalizing ankle and knee angles and moments which requires adequate AFO stiffness (Ploeger et al., 2019; Waterval et al., 2019). Normalization of the ankle and knee biomechanics is hypothesized to lead to a decrease in the metabolic cost of the quadriceps muscles and thereby reduce mCoT (Kobayashi et al., 2017). In this context normalization means that the ranges of angles and moments are brought closer to the “normal” angle and moment range of a healthy individual during walking. The initial decrease in mCoT may be further explained by a reduction in the metabolic cost of the plantar flexors as the AFO replaces the biological ankle plantarflexion moment during stance (Bregman, 2011; Arch et al., 2016; Sreenivasa et al., 2017). However, at higher stiffnesses, as the AFO restricts the ankle range of motion (RoM) (Bregman, 2011; Harper et al., 2014; Ploeger et al., 2019), it limits active biological ankle power generation and energy storage and release (Bregman, 2011; Bregman et al., 2011) of the AFO during push-off (Ploeger et al., 2019; Waterval et al., 2019). The reduced ankle push-off work may result in higher energy losses at contralateral heel-strike and lead to compensatory hip flexion work to initiate the swing phase (Bregman, 2011), which are potential causes for the increased mCoT at higher AFO stiffness levels. However, how each of these factors contribute to the relation between AFO stiffness and mCoT in people with plantar flexor weakness is unknown.

The aim of this study was to gain insights into how mCoT is affected by AFO stiffness variation in individuals with plantar flexor weakness by using predictive musculoskeletal simulations. We tested whether initial reductions in mCoT with increasing stiffness are explained by i) decreasing metabolic cost of the quadriceps as the knee moments are normalized, and ii) decreasing metabolic cost of the plantar flexors as the AFO replaces the ankle plantar-flexion moment during stance phase. Third, we hypothesized that increases in mCoT as stiffnesses exceed the optimum stiffness are caused by the increasing metabolic cost of hip flexor muscles to initiate the swing phase as total push-off power decreases.

## 2 Materials and methods

We created a planar musculoskeletal model of an individual with bilateral plantar flexor weakness, similar to (Waterval et al., 2021) using OpenSim (Delp et al., 2007; Seth et al., 2018), and implemented an AFO with varying stiffness. To generate predictive gait simulations, we employed a reflex-based neuromuscular controller and optimized the control parameters using dynamic optimization to minimize mCoT, and solved the optimization problem in SCONE (Geijtenbeek, 2019; Ong et al., 2019; Waterval et al., 2021). Predictive gait simulation results were compared to experimental data of subjects with bilateral plantar flexor weakness walking with varying AFO stiffness (Waterval et al., 2019).

### 2.1 Musculoskeletal model

Based on the model of Delp et al. (Delp et al., 1990; Ong et al., 2019), we created a model with 9 degrees of freedom (3 around the pelvis and one around the hip, knee and ankle of each leg), actuated by 9 Hill-type muscles per leg (tibialis anterior, soleus, gastrocnemius, vasti, rectus femoris, biceps femoris short head, biarticular hamstrings, iliopsoas, gluteus maximus) in OpenSim 3.3 (Delp et al., 2007; Seth et al., 2018). We set the maximum isometric muscle strength of the soleus and gastrocnemius muscles of both legs to 40% of normal healthy values (i.e. 60% muscle weakness), to induce bilateral plantar flexor weakness. Additionally, we restricted the ability to activate the plantar flexor muscles to 50%, to take into account that the weakened muscles would completely fatigue if they would be maximally activated for 10%–20% of the gait cycle (Bigland-Ritchie et al., 1986; Schillings et al., 2007; Potvin and Fuglevand, 2017). We modified passive muscle and tendon parameters in the model to maintain similar passive muscle forces as in the healthy model (Umberger et al., 2003; Waterval et al., 2021). We set the slow twitch fiber ratios according to Johnson et al. (Johnson et al., 1973) and Garrett et al. (Garrett et al., 1984), similarly to Ong et al. (Ong et al., 2019). We scaled the model according to experimental marker data of one subject with bilateral plantar flexor weakness close to the group’s mean height (177 cm) and body mass (81 kg) from the experimental study (Waterval et al., 2019).

**FIGURE 1:**
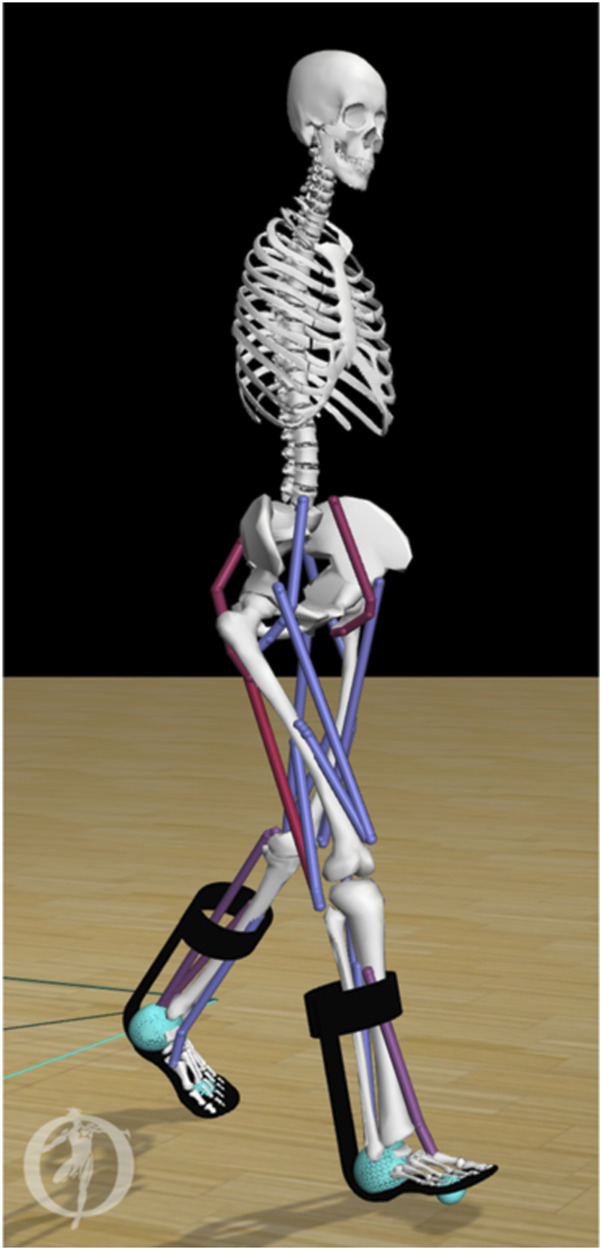
Musculoskeletal model of an individual with bilateral plantar flexor weakness equipped with AFOs. The stiffness (range 0–7 Nm/deg) of the dorsal leaf spring is modeled in OpenSim as a torsional spring between the calf-casing and footplate of each AFO (black). Contact between the foot and the ground are modeled by forces generated by compliant contact spheres (cyan).

To model the forces between the ground and the foot, we used a compliant Hunt-Crossley contact model (Sherman et al., 2011). We placed one contact sphere at the heel and one at the toe of each foot (Figure 1). We set the force parameters (stiffness, dissipation and friction) according to Veerkamp et al. (Veerkamp et al., 2021), and modeled the knee ligaments using a rotational spring (2 Nm/deg) and damper (0.2 Nm/deg/s) around the knee joint if the knee angles were outside the 5–120 deg flexion range (Waterval et al., 2021).

We modeled each AFO as two rigid parts, including a calf casing and a footplate with their experimental mass (calf casing: 0.2 kg, footplate and shoe: 0.5 kg) (Waterval et al., 2019). We attached the AFO parts rigidly to the tibia and calcaneus, respectively (Figure 1). We modeled the stiffness of the AFO as one linear torsional spring for ankle dorsiflexion and one linear torsional spring for plantarflexion with the same stiffness. In order to match experimental movement of the ankle within the AFO, the AFO did not deliver a moment in the neutral angle range, i.e., between 4.5 deg plantarflexion and 2 deg dorsiflexion. In DLS-AFOs, this small range depends on the material and manufactured geometry of the AFO, and its fit on the subject’s leg. The neutral angle range was defined from the ankle angle range during swing phase of the subject because in swing phase the AFO exerts only small moments on the ankle joint (Ries and Schwartz, 2019).

### 2.2 Simulation framework

We used SCONE (v1.6.0), a simulation, control, and dynamic optimization framework (Geijtenbeek, 2019), to simulate gait of 10 s in duration. The muscle activations were generated by a reflex-based gait controller (Geyer and Herr, 2010; Ong et al., 2019), whose parameters were optimized by minimizing the specified objective function using the Covariance Matrix Adaptation evolutionary strategy (CMA-ES) (Hansen, 2007; Song and Geyer, 2015; Ong et al., 2019; Waterval et al., 2021).

The objective function (*J*) was comprised of desired high-level tasks during gait, where the goal was to minimize mCoT (J_mCoT_), high loading rates at impact (*J*_FGImpact_), and head accelerations (*J*_HeadStab_). Only optimizing towards energy cost does result in human-like gait but has some limitations. We incorporated the other functions that are usually used to improve simulations of healthy gait, as in humans these also play a role (Veerkamp et al., 2021). The objective function including the same additional objectives as we used, was validated to predict the effects of bilateral plantar flexor weakness on gait in a previous predictive simulation study (Waterval et al., 2021). Penalties were defined to keep the model walking faster than a minimum velocity and to keep passive forces in the knee and ankle within physiological limits.

J_mCoT_ was the mCoT measure, which aggregated the total muscle metabolic cost divided by the distance travelled. We computed the metabolic cost of each individual muscle, according to the muscle metabolic model by Uchida et al. (Uchida et al., 2016).

*J*_FGImpact_ was a measure composed of the sum of the absolute ground reaction force derivative over the simulation divided by the distance traveled, which was included to minimize high loading rates at impact. High loading rates were minimized because they have been proposed to contribute to injuries by applying high stresses to the legs (Nigg, 1985; Mündermann et al., 2005).

*J*_HeadStab_ was a measure for excessive head accelerations calculated as the sum of the absolute head accelerations normalized by distance traveled (Pozzo et al., 1990; Bril and Ledebt, 1998). Minimizing head accelerations is frequently used in simulation studies (Dorn et al., 2015; Ong et al., 2019), and it comes from the finding that a primary objective of the postural control system is to keep the head stable during different walking conditions (Menz et al., 2003).

*P*_Gait_ penalty was added to the objective function to keep the model walking at least with the specified minimum velocity of 1.22 m/s without falling down. The chosen minimal velocity was the minimal velocity of the patient in the experimental study (Waterval et al., 2019), whose attributes were used for scaling the model, since walking speed and leg length are related (Alexander, 1989). The minimal velocity value was the same in all optimizations, it was not used to match experimental velocities.

We added *P*_DOFLimAnkle_ and *P*_DOFLimKnee_ penalties to keep the ankle angle and passive knee forces within physiological limits. We gave penalties, when the ankle angle was outside of the [−60, 60] deg range and when the absolute coordinate limit moment acting on the knee joint was larger than 5 Nm (Waterval et al., 2021).

The weights associated with these high-level tasks were w_mCoT_ = 1.5, w_FGImpact_ = 0.05, w_HeadStab_ = 0.1, w_Gait_ = 10^9^, w_DOFLimAnkle_ = 0.1, w_DOFLimKnee_ = 0.01. We chose the weights based on a previous study (Waterval et al., 2021), but adapted with a higher emphasis on *J*_mCoT_ to capture its effect because our goal is to explain the differences in mCoT between stiffness levels. We ran the simulations for stiffness levels between 0 and 7 Nm/degree, with steps of 1 Nm/degree. We ran five optimizations in parallel with different random seeds in each round. Each optimization was terminated when the average reduction of the cost function score in the last 200 generations was smaller than 0.01%. As initial guess, we used a controller with parameters resulting in healthy gait (Waterval et al., 2021). We set the initial step size (σ), as the initial standard deviation of the parameters, to 0.05 (Ong et al., 2016). The number of optimization parameters was 100 in our optimization which is the dimension (n) of the problem. This contains 89 parameters for the variables of the gait controller, 4 parameters for the gait phase transition thresholds, and 7 parameters for the initial state, defining the joint position offsets. The population size was set to 17, calculated according to the recommendation from Hansen (2007).

To check the robustness of our results, we ran multiple optimizations in sequence. We used the results with the smallest objective function value to initialize the next optimization with the same model (same AFO stiffness setting) and the same initial step size, similar to Song and Geyer (2015) and Ong et al. (2016). Since the trend of the results was not changing qualitatively between the first and second round of optimizations, we performed only two rounds of optimizations. We considered the results of the second round as the final results.

### 2.3 Comparison with experimental data

To test the goodness of the gait change predictions with varying AFO stiffness, we compared our predictive gait simulations with experimental gait and mCoT data of 24 bilateral weakness subjects walking with five different AFO stiffness configurations (2.8, 3.5, 4.3, 5.3, 6.6 Nm/deg) (Waterval et al., 2019). In this experimental study, the mean mCoT (in J/kg/m) was evaluated from a 6-min comfortable walk test with simultaneous breath-by-breath assessment of oxygen consumption (VO2) and carbon dioxide production (VCO2) over the last 3 min of the test. The study assessed gait kinematics, kinetics and ground reaction forces with a 3D gait analysis on a 10-m walkway using the PlugInGait marker model (Vicon Motion Systems, 2019). Based on these measurements, clinically important gait features for the evaluation of AFOs, e.g., the peak ankle dorsiflexion angle in stance, ankle power in stance, knee angle in stance, external knee extension moment in late stance (between 35%–50% of the gait cycle) and AFO-generated power in stance, were calculated using a custom-made script in MATLAB^®^ R2015b (MathWorks Inc.). Further biomechanically relevant data of the subjects and experimental details are available in (Waterval et al., 2019).

To calculate the joint moments from the predictive gait simulations, we processed the simulation results with the Analysis Tool in OpenSim. Based on the joint angles and moments, we calculated the joint powers. To calculate negative and positive joint work over the whole gait cycle and separate gait phases we used trapezoidal numerical integration of the joint power. We divided the gait cycle into gait phases according to the definitions of Whittle (Whittle, 2007). These gait phases were: loading response, midstance, push-off consisting of terminal stance and preswing, and swing. In order to assess the source of the mCoT in detail, we calculated the simulated muscle metabolic energy cost (Uchida et al., 2016) over whole gait cycles, and different gait phases, for all nine muscles. We normalized the metabolic cost by body mass, mean walking speed and simulation duration to get the total metabolic cost over a gait cycle in J/kg/m. We used the averaged data of all full gait cycles after the first two gait cycles in the simulation result for the analysis of a gait cycle. We used a custom-made script in MATLAB^®^ R2020b (MathWorks Inc.) for all calculations.

To compare predictive gait simulation outcomes to experimental observations, we calculated the effect of one additional Nm/degree in stiffness for both the simulations and experimental data using a linear fit across the stiffness levels for the following key gait features: peak ankle dorsiflexion angles, peak total-, biological- and AFO ankle joint moments and powers, peak knee extension angles and peak knee joint moments (between 35%–50% of gait cycle) (Waterval et al., 2019). We assessed the goodness of fit of the curve by its coefficient of determination value (Rsq), calculated in MATLAB^®^ R2020b (MathWorks Inc.) (MathWorks, 2016). To assess the similarity between the simulated and experimentally obtained slopes, we expressed the difference in slope in standard deviation of the experimental slopes based on the 24 patients. The standard deviation of the experimental slope shows how dispersed the experimental slopes are in relation to the mean experimental slope. By expressing the difference of the mean experimental slope and the simulated slope in standard deviation of the experimental slope, it shows if the difference would be in the range of the experimental data variability. If the simulated data is in the range of variability (within one standard deviation) of the experimental data, then the simulated data could realistically represent an instance of the experimental data.

## 3 Results

**FIGURE 2:**
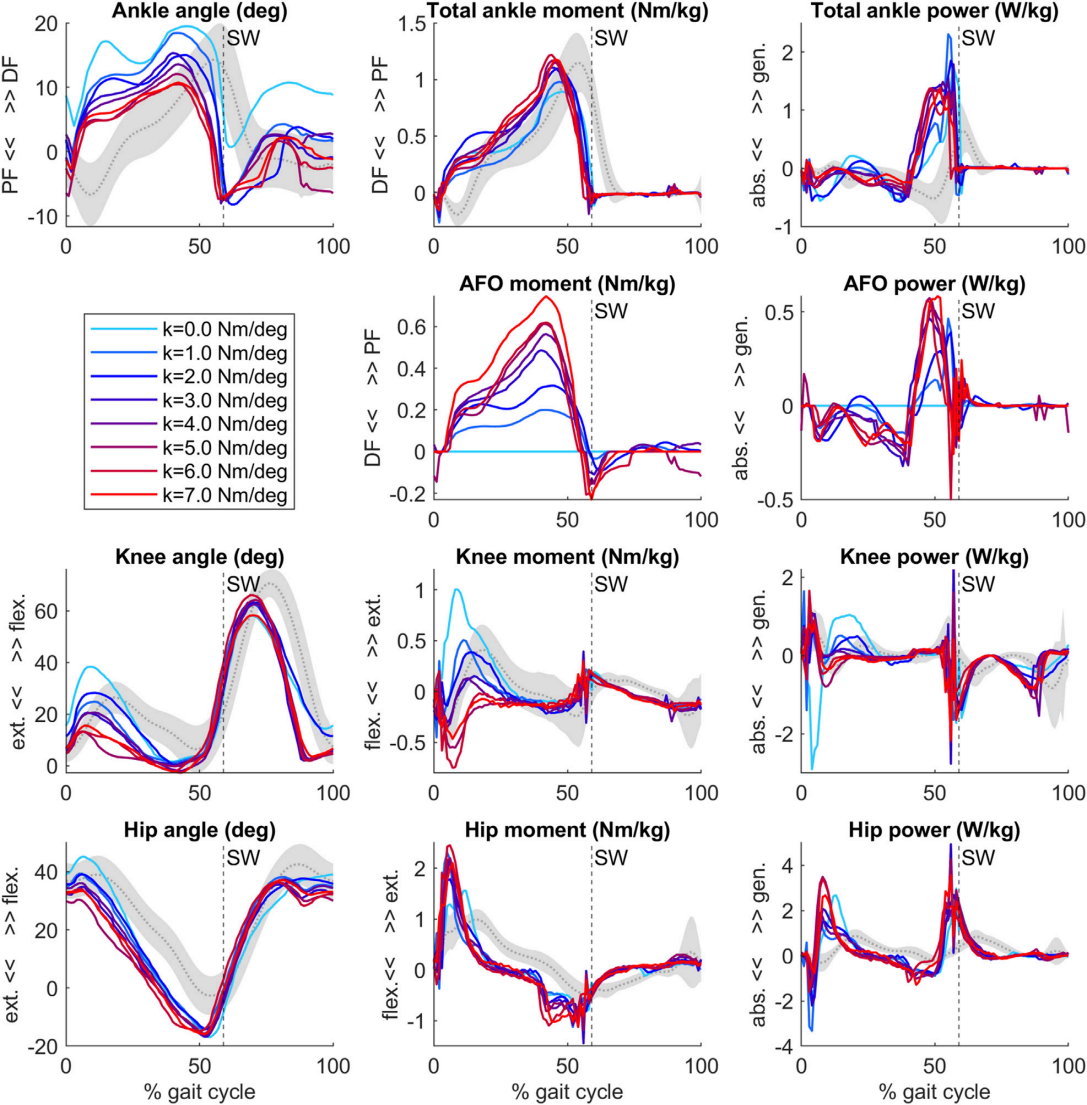
Ankle, AFO, knee and hip angles, internal moments and powers from the predictive gait simulations. Simulation data of an individual patient (Waterval et al., 2019) model with bilateral plantar flexor muscle weakness wearing an AFO with varying stiffness levels (range 0–7 Nm/deg, colored lines). Gray curves and shading represent patient data without AFO (mean ± 1 SD) (Waterval et al., 2019). Vertical dashed line with SW text marks the beginning of swing. After initial contact, the ankle, knee and hip joints became more extended as AFO stiffness increased.

Our predictive gait simulations took 20.46 h on average to complete on an AMD Ryzen 9 3950X (16 CPUs—32 virtual cores with hyperthreading, 3.5 GHz base) computer on 10 parallel threads. An overview of the simulated joint angles, moments and powers for varying AFO stiffness levels are presented in Figure 2.

### 3.1 Comparison of simulated and experimental results

**FIGURE 3:**
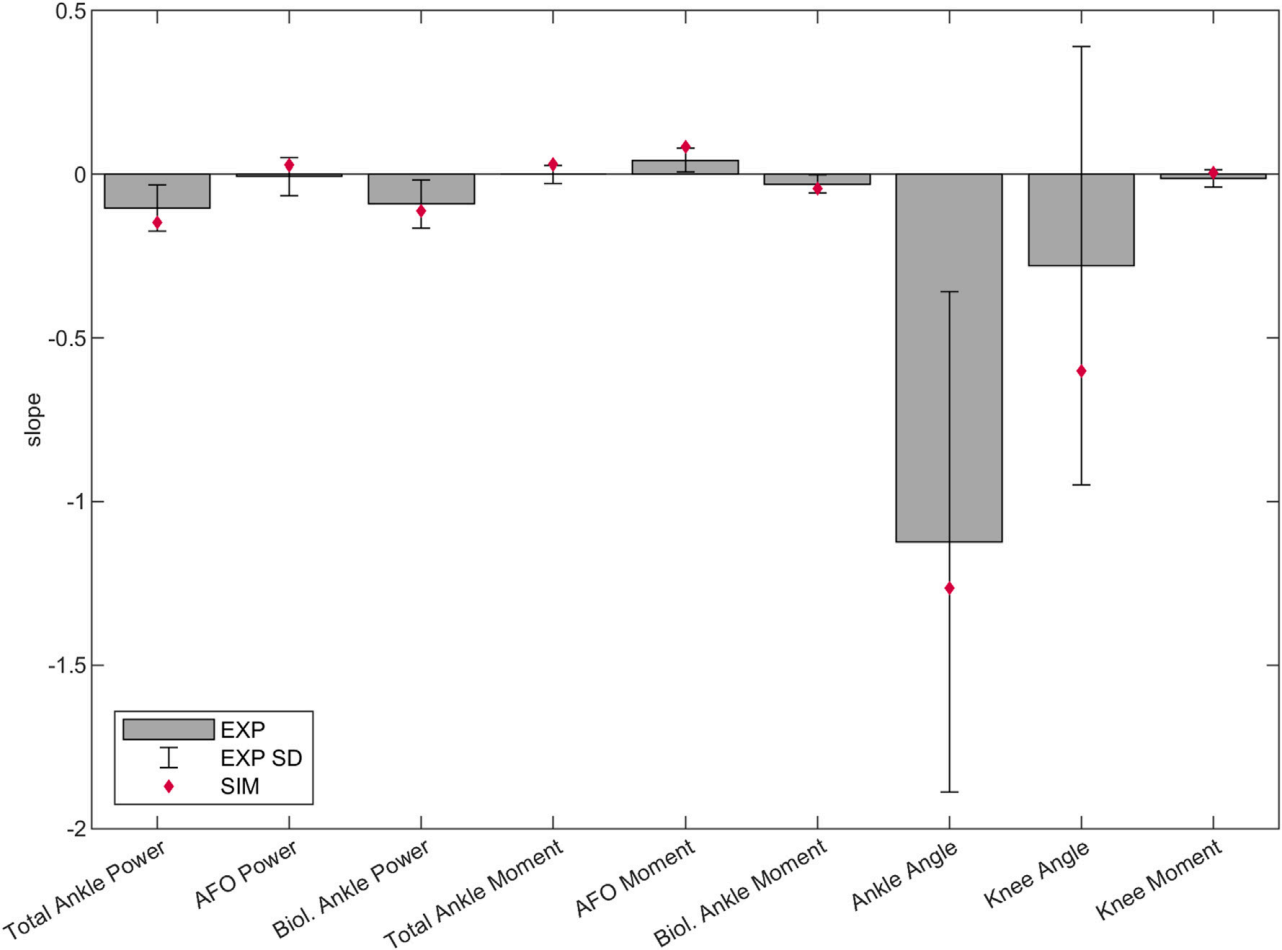
Predicted, and mean and SD of experimental slopes of peak total, biological and AFO-provided ankle joint power and internal moment in stance, peak ankle dorsiflexion angle in stance, peak knee extension angle in stance and peak internal knee flexion moment in late stance (between 35%–50% of the gait cycle). Lines were fitted to the individual experimental and predictive gait simulation data across 1–7 Nm/deg AFO stiffness levels. The bars and the error bars represent the mean and SD of the slope of the lines fitted to the individual experimental data of the 24 patients (Waterval et al., 2019). The diamond shaped markers show the slope of the lines fitted to the predictive gait simulation data. Negative and positive directions are defined the same as in Figure 2. Negative slope means change into absorption direction by ankle/AFO powers, change into internal dorsiflexion moment direction by ankle/AFO moments, change into plantarflexion angle direction by ankle angles, change into knee extension direction by knee angles, and change into knee flexion moment direction by knee moments. The slope, SD and goodness of fit (Rsq) values can be found in the Supplementary Table S1.

The predicted slopes of peak total, biological and AFO-provided ankle joint moment and power, peak ankle dorsiflexion angle, peak knee extension angle and peak internal knee flexion moment were all within 1.2 standard deviations (SD) of the experimental data. The goodness of fit (Rsq) values of the slopes can be found in Supplementary Table S1. The highest slope difference was found for peak total ankle moment and peak AFO moment, where a larger effect of additional stiffness was predicted by the simulations than found in experimental data. Peak total ankle moment was approximately constant in the experiments but showed an increasing trend in the predictive gait simulations (Figure 2; Figure 3, Supplementary Table S1, S5).

### 3.2 Simulated AFO effects on mCoT and muscle metabolic consumption

**FIGURE 4:**
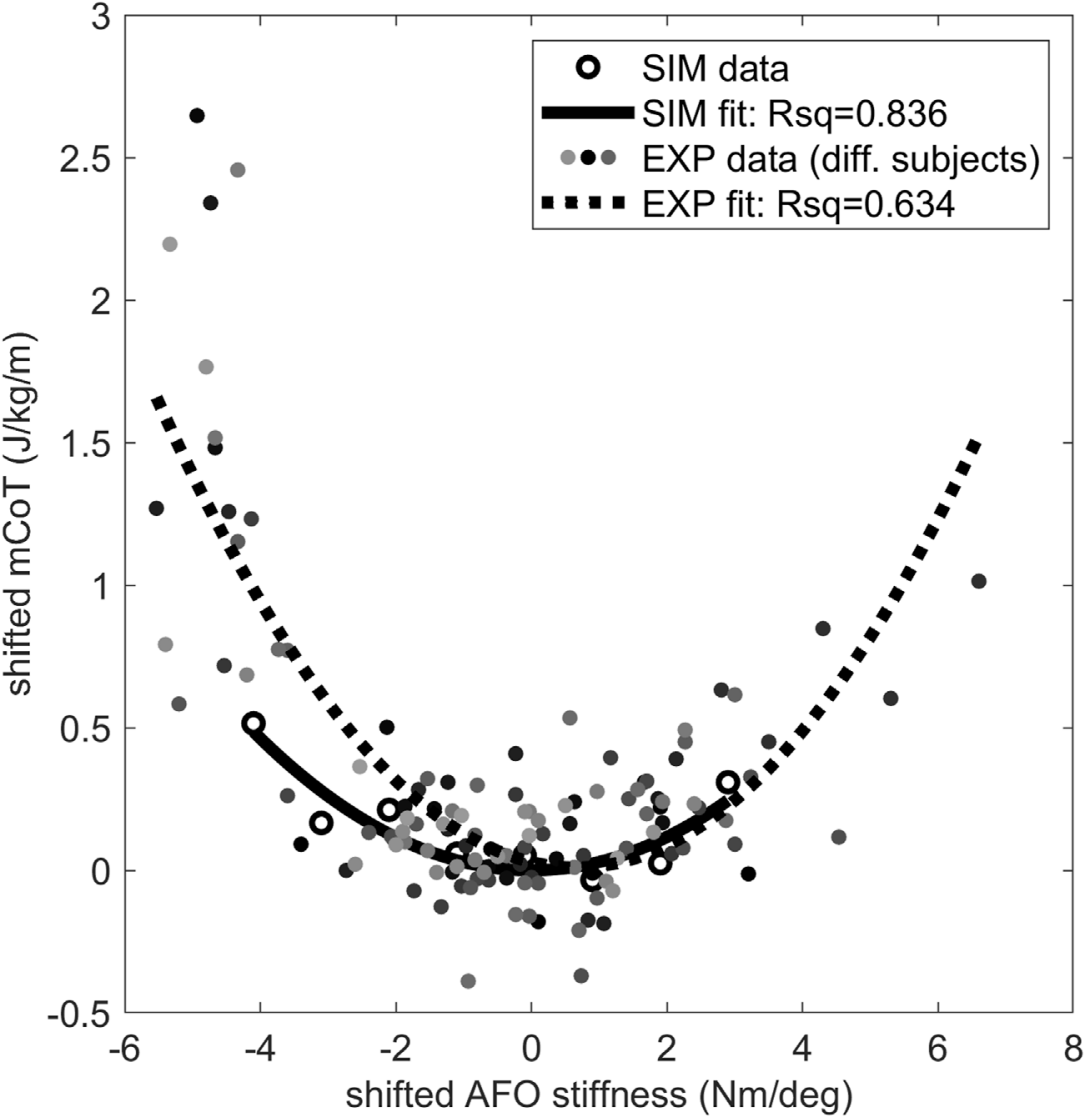
The mCoT in predictive gait simulation and individual experiments wearing an AFO with varying stiffness. The individual experiments included the condition without an AFO and wearing shoes for 21 subjects (Waterval et al., 2019). A quadratic curve was fitted to the data points for each subject dataset and the minimum of the fitted curve was taken for each subject as their individual minimum mCoT value which is happening at their individual optimal stiffness. The mCoT values of the subjects were shifted by their minimum mCoT value and the AFO stiffness values were shifted by the subject’s optimal stiffness value. The shifted individual experimental subject data is shown in different shades of gray. One quadratic curve was fitted to all shifted experimental subject data (dotted line), the goodness of fit is indicated by the Rsq number on the plot (Rsq = 0.634). The same was done for the predictive gait simulation results (solid line, Rsq = 0.836). Both the experimental (EXP–shaded dots) and predictive gait simulation data (SIM–open circles) show quadratic trends (Rsq >0.63).

The mCoT showed a clear minimum with increasing stiffness in both the simulated and individual experimental results (Figure 4). The predictive gait simulations presented a strong quadratic trend, Rsq = 0.836, while the average experimental results showed a less pronounced quadratic trend, Rsq = 0.634, due to large inter-subject variability (Figure 4).

**FIGURE 5:**
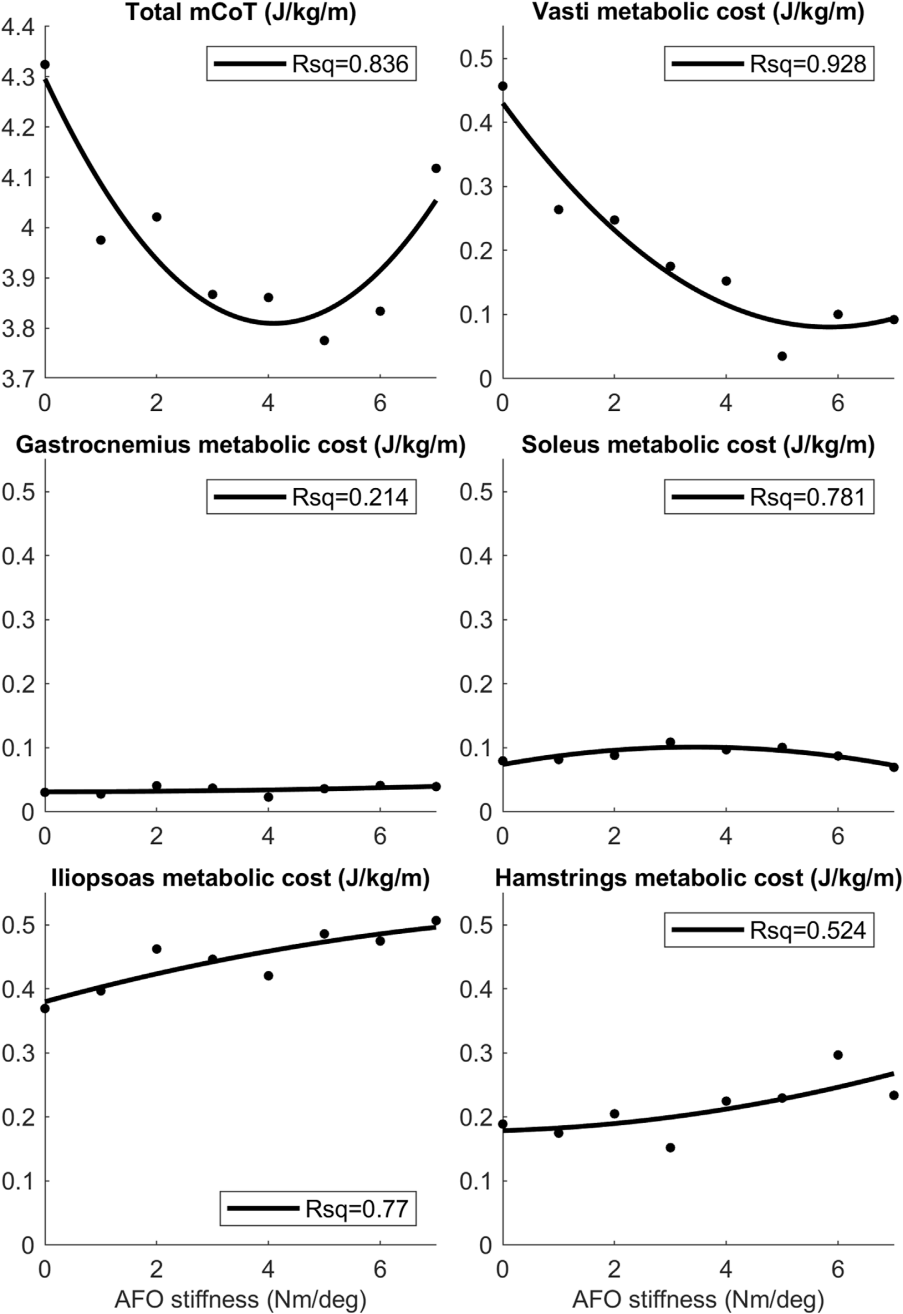
The mean mCoT, and mean metabolic cost of the top five muscle contributors in the predictive gait simulations. Metabolic cost data for the mean was taken during one whole gait cycle as AFO stiffness was varied from 0–7 Nm/deg. Quadratic curves were fitted to the data-points and the Rsq values represent the goodness-of-fit of the curves. Values of the mean metabolic cost for the whole model and for each muscle group of the model can be found in the S2 Table.

The largest change in metabolic cost of individual muscles was found in the vasti, which also showed a quadratic trend (Rsq = 0.928). In contrast, the metabolic cost of the hamstrings and iliopsoas increased continuously. Both the soleus and gastrocnemius metabolic cost did not change substantially with increasing AFO stiffness (Figure 5 and Supplementary Table S2). Gluteus maximus, tibialis anterior, rectus femoris and biceps femoris short head muscles did not show any change with increasing AFO stiffness (Supplementary Table S2).

### 3.3 Simulated AFO effects on muscle metabolic consumption and joint work per gait phase

**FIGURE 6:**
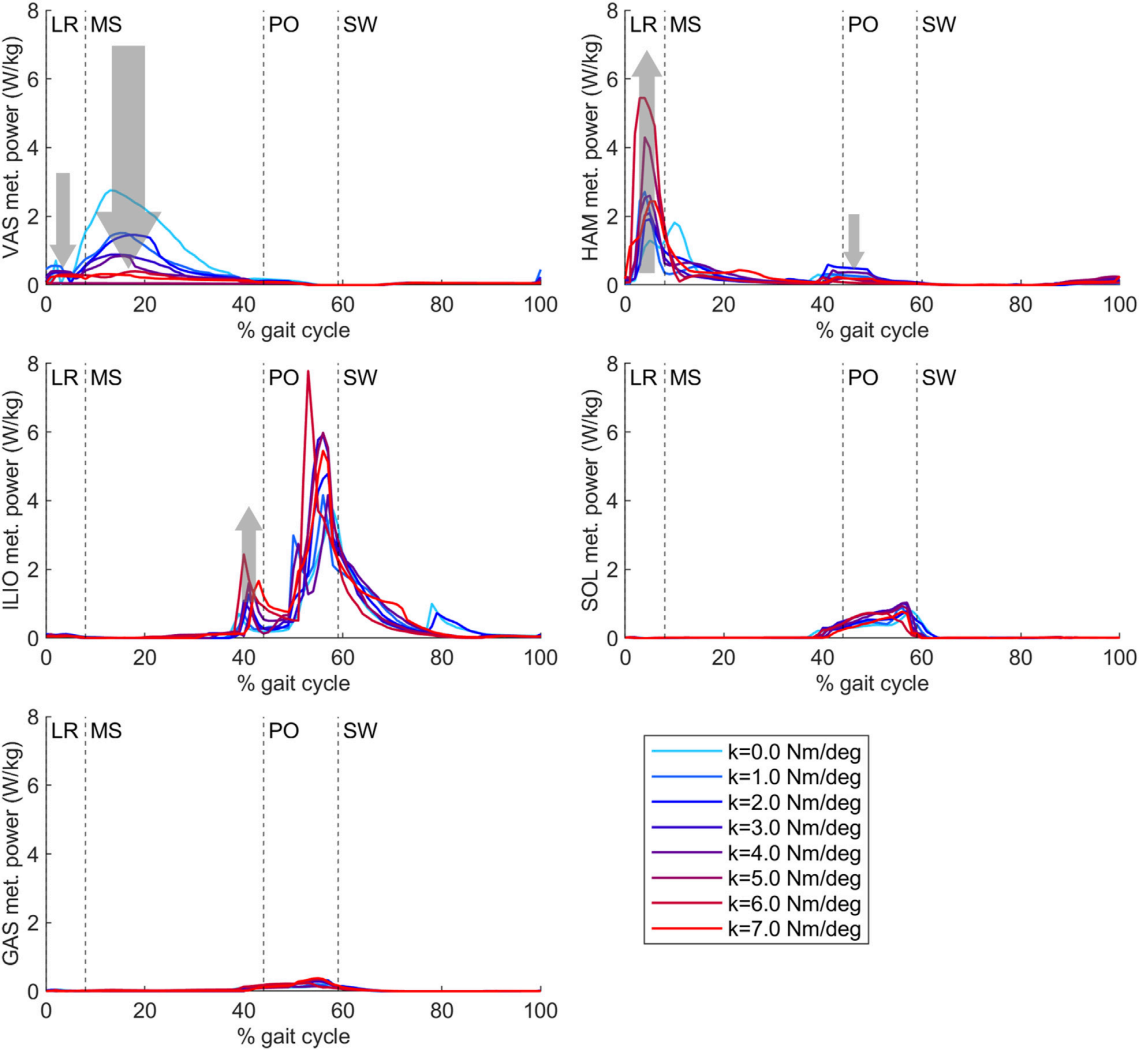
Vasti, hamstrings, iliopsoas, soleus and gastrocnemius metabolic power from the predictive gait simulations. AFO stiffness was varied from 0 to 7 Nm/deg. Abbreviations: VAS: vasti muscle group, HAM: hamstrings muscle group, ILIO: iliopsoas muscle group, SOL: soleus muscle, GAS: gastrocnemius muscle, LR: loading response, MS: midstance, PO: push-off, SW: swing, k: AFO stiffness. The gray arrows show the change direction in metabolic cost as AFO stiffness was increased. The size of the arrows is in approximate relation to the size of the metabolic cost changes. The metabolic cost of transport values of each muscle group calculated with integration for each gait phase can be found in Supplementary Table S4.

Total knee joint work did not differ with increasing AFO stiffness during the loading response, while vasti metabolic cost decreased and hamstrings metabolic cost increased, especially above 3 Nm/degree. In midstance, positive knee joint work decreased, negative knee joint work increased and vasti metabolic power decreased, while hamstrings metabolic power did not show a clear trend with increasing AFO stiffness. (Figure 6, Supplementary Figure S2 and S3, Supplementary Tables S3 and S4).

During loading response, negative biological ankle work decreased with increasing AFO stiffness, while no effect of stiffness on AFO work was found. Similarly, no effect on the metabolic cost of the soleus or gastrocnemius was found with increasing stiffness. In midstance, negative biological ankle joint work decreased and negative AFO work increased with increasing stiffness. Soleus metabolic cost increased slightly until 5 Nm/deg AFO stiffness. During push-off, biological ankle work, AFO work, and soleus metabolic cost increased until 3 Nm/deg. At higher stiffnesses, biological ankle work generation and soleus metabolic cost decreased again (Supplementary Figures S2 and S3, Supplementary Tables S3 and S4).

During loading response, negative hip joint work decreased and hamstrings metabolic cost increased with increasing stiffness. In early midstance, positive hip joint work increased, and in late midstance, negative hip joint work and iliopsoas metabolic cost increased with increasing AFO stiffness (Figure 6, Supplementary Figures S2 and S3, Supplementary Tables S3 and S4).

## 4 Discussion

The aim of this study was to gain insights into how motor pattern adaptations in people with bilateral plantar flexor weakness result in an optimal AFO stiffness to minimize mCoT. As hypothesized, initial reductions in mCoT with increasing stiffness were attributed to reductions in quadriceps metabolic cost, but in contrast to our hypothesis, plantar flexor metabolic cost did not decrease. Increases in mCoT above the optimum AFO stiffness were attributed to the increasing metabolic cost of both hip flexor muscles and hamstrings muscles.

Our predictive gait simulations predicted changes in lower extremity kinematics and kinetics due to AFO stiffness variations within 1.2 SD of the experimentally observed changes. Differences between simulated and experimental data were found in the knee joint angle and moment curves (Figure 2). Although the effects of varying AFO stiffness on specific gait features at late stance were predicted reasonably well (Figure 3), in early stance the knee angle became more extended (reduced flexion) and the internal knee moment changed from extension moment into flexion moment with increasing AFO stiffness (Figure 2). This effect is likely explained by the fact that more extended knees in early stance are linked to decreased mCoT (Waters and Mulroy, 1999; Brehm et al., 2007; Brehm et al., 2008). The human likely minimizes or is subjected to other factors, such as muscle fatigue and loading rate (Crowninshield and Brand, 1981; Ackermann and van den Bogert, 2010; Veerkamp et al., 2021; Waterval et al., 2022), which may cause the knee flexion in loading response instead of the metabolically more efficient straight knee. Although loading rates were penalized in our predictive gait simulations, loading rates still increased up to twice as much as found in healthy subjects as AFO stiffness increased (Supplementary Figure S1), which could increase the risk of lower limb stress fractures (Cook et al., 1997; Zadpoor and Nikooyan, 2011).

The convex mCoT trend with respect to AFO stiffness can be explained by the metabolic cost changes in the quadriceps (vasti), hip flexor (iliopsoas) and hamstrings muscles. This parabolic trend was also present in the individual experimental data (Waterval et al., 2019) (Figure 4). As hypothesized, initial reductions in mCoT starting at low and with increasing AFO stiffness were due to a decrease in the metabolic cost of the quadriceps (vasti) muscles (Figure 5). From low to medium AFO stiffnesses, the knee angle and moment normalized, reducing the metabolic cost of the vasti (Waters and Mulroy, 1999; Brehm et al., 2008). At higher stiffness levels, the knee became increasingly extended, which minimized mCoT but might cause knee pain in real life (Cook et al., 1997; Zadpoor and Nikooyan, 2011). Contradicting our hypothesis and experimental data in healthy subjects (Collins et al., 2015), metabolic cost of the plantar flexor muscles did not decrease with increasing AFO stiffness. As muscle activation and metabolic cost changes are related factors, our predictive gait simulation result was also contrary to the findings of Harper et al. (Harper et al., 2014) who found reductions in medial gastrocnemius muscle activation with increasing AFO stiffness in patients with lower limb impairments (Harper et al., 2014). We likely did not observe reductions in plantar flexor metabolic cost, because the low muscle strength in the model resulted in proportionally low muscle mass, which reduces the muscle’s contribution to metabolic cost (Umberger et al., 2003), and hence, even without an AFO the plantar flexors did not contribute substantially to mCoT.

In agreement with our hypothesis, increases in mCoT above the optimum stiffness were partly due to increases in the metabolic cost of the hip flexor (iliopsoas) muscles. Iliopsoas metabolic cost increased at the end of midstance before the start of push-off, potentially as a pre-activation to help initiate the swing phase. Increased hip work was also shown in an experimental study in patients with chronic stroke or multiple sclerosis where 0.5–5.4 Nm/deg AFO stiffnesses were tested (Bregman, 2011). Additionally, an increase in metabolic cost of the hamstrings muscles added to the increase in mCoT above the optimum stiffness. This metabolic cost increase can be seen during early stance where slightly more extended hip joint angles, larger hip extension moments and decreasing negative hip joint work (Supplementary Figure S3) can also be observed as AFO stiffness increases. At high stiffness levels, the hip is more extended at initial contact, and hip flexion is reduced during loading response that further contributes to the increased knee joint loading rates at high stiffness.

To test our hypotheses, we used a simplified, planar musculoskeletal model where medio-lateral stabilization was excluded, which could explain why our mCoT results were ∼10% lower (Matsubara et al., 2015) than in the experiments. With suboptimal AFO settings, the out-of-plane compensation, such as trunk motions, are known to be more extreme (Meyns et al., 2020), hence the sensitivity of the mCoT trend to AFO stiffness could be higher in reality than in our predictive gait simulations. However, most of the gait changes with AFO use on patients with plantar flexor weakness occur in the sagittal plane (Waterval et al., 2019; Waterval et al., 2023). Additionally, Donelan et al. showed that providing external lateral stabilization to subjects walking on a treadmill with their preferred step width reduced their metabolic cost only by 5.7% (Donelan et al., 2004). Consequently, as sagittal plane muscle actions contribute most of the total metabolic cost (Donelan et al., 2004) and most of its adaptations to AFOs (Waterval et al., 2019; Waterval et al., 2023), our goal to explain the convex relation between mCoT and AFO stiffness can be done with a planar model.

The reliability of the model’s plantar flexor muscle properties is uncertain. Peak AFO moment increases more with AFO stiffness in the predictive gait simulation than in the experimental data (Figure 3). Consequently, peak total ankle moment also increases more with AFO stiffness. As peak AFO moment comes from AFO stiffness and the peak ankle angle, and AFO stiffness is the same in the comparison plot (Figure 3), peak angle is the one that increases more in the predictive gait simulation than in the experiments. This could be, for example, due to stiffer muscles around the ankles of the patients than in the model’s muscle properties. This influences the stiffness ratio between the biological muscle and the AFO and could enhance the effect of the AFO in the predictive gait simulations.

In the experimental study that was used for comparison, only AFOs with a stiffness in the range of 2.8–6.6 Nm/deg were tested. Hence, we were unable to verify the validity of our prediction with low stiffness levels.

Limitations of the optimization framework include the non-convexity of the optimization problem and the stochasticity of the optimizer, which means that the predictive gait simulation results are unlikely to be global minima (Ong et al., 2019). Following previous studies (Song and Geyer, 2015; Ong et al., 2016), we restarted optimizations from the previous best result to gain confidence in our solutions.

The purpose of the objective function is to reflect the goals of human walking that are relevant to our research goals. To identify the most appropriate configuration, we conducted several preliminary experiments to assess the impact of various objective functions. Nevertheless, it is possible that other unconsidered factors might also play a role such as robustness and tolerance of motor and sensory noise (Van Wouwe et al., 2022). Additionally, the weights assigned to each term might be inaccurate, as the goals of human gait vary from person to person, particularly among those with pathological conditions, complicating the process of precise adjustment.

In the future, simulations might be used to predict the individual optimal AFO properties. In this study, we demonstrated how predictive gait simulations can help us understand the underlying mechanisms of reduced energy expenditure with a tuned AFO as an essential first step. To be able to accurately predict adaptations to an AFO at the level of an individual, out-of-plane degrees of freedom and muscle actions should be investigated to understand the effects of out-of-plane compensations (Song and Geyer, 2013). Furthermore, sensitivity analyses should be performed to evaluate the effect of motor and sensory noise, patient and device characteristics, such as body weight, muscle weakness and muscle spasticity, the neutral angle range of the AFO, and patient reported outcomes on the optimal AFO stiffness (Waterval et al., 2020; Waterval et al., 2023). With individualized models, our predictive gait simulations could help predict the individual adaptations of patients to an AFO and improve the prescription of AFO settings (Falisse et al., 2019).

Our predictive gait simulation results demonstrate the convex relation between mCoT and AFO stiffness, and are able to explain this shape by decreases in quadriceps metabolic cost in midstance, and increases in metabolic cost of the hamstrings during loading response and iliopsoas in mid-to-late stance as AFO stiffness increases above the optimal. In the future, mCoT minimization may enable predictions for individualized gait adaptations to an AFO for people with bilateral plantar flexor weakness and facilitate optimal AFO prescriptions. The musculoskeletal models (in OpenSim, https://simtk.org/projects/opensim) and code, which were used to execute the gait optimization (in SCONE, https://simtk.org/projects/scone), and our complete predictive gait simulation results are provided at https://simtk.org/projects/afo-predictions.

## Data Availability

The datasets presented in this study can be found in online repositories. The names of the repository/repositories and accession number(s) can be found in the article/Supplementary Material. The data is available at: https://simtk.org/projects/afo-predictions.
