# Supplementary figures and images for: Minimization of metabolic cost of transport predicts changes in gait mechanics over a range of ankle-foot orthosis stiffnesses in individuals with bilateral plantar flexor weakness
Source: Front Bioeng Biotechnol. 2024 May 23;12:1369507. doi: 10.3389/fbioe.2024.1369507 (PMC11153850; doi:10.3389/fbioe.2024.1369507)

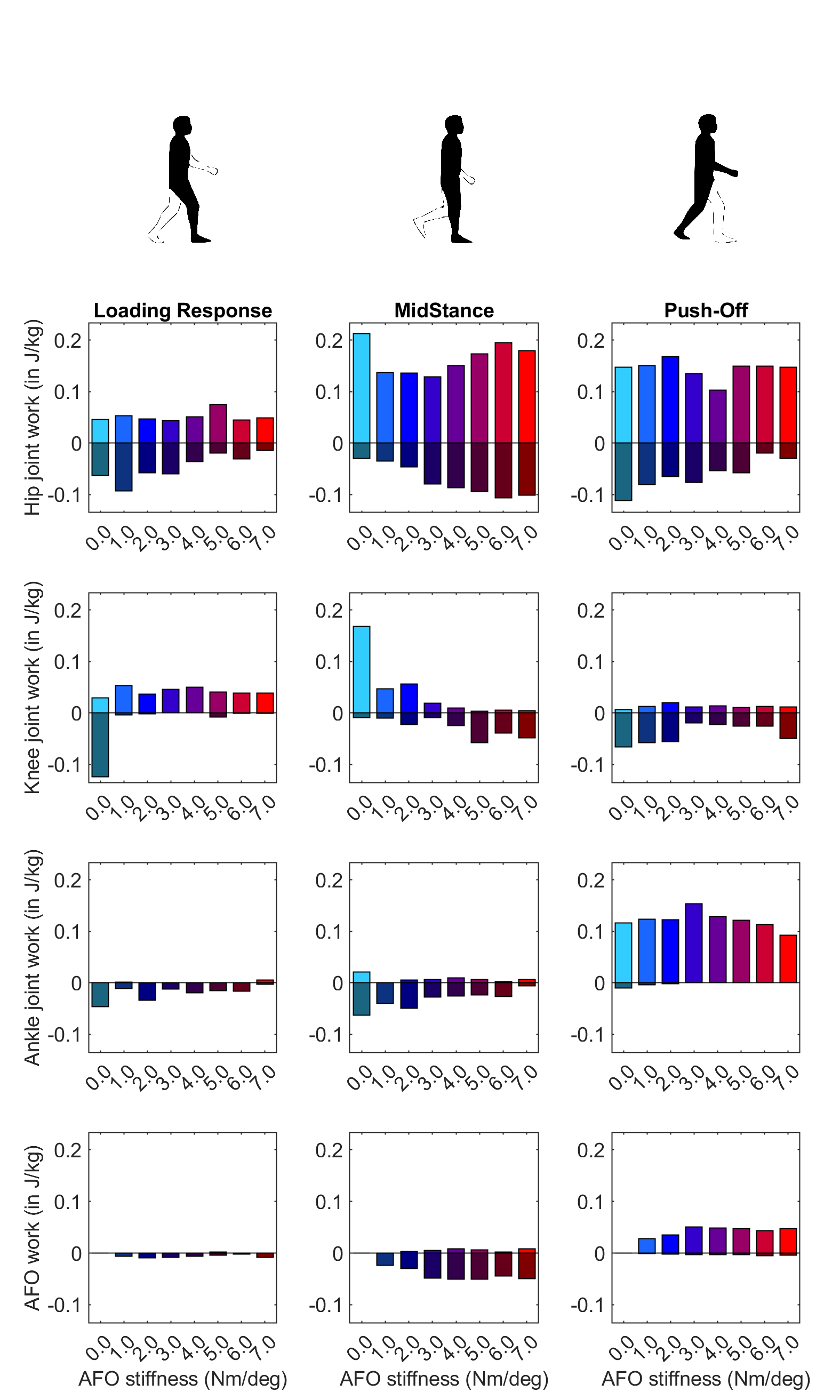

Supplement: Supplementary file 1 [file Image3.TIFF]

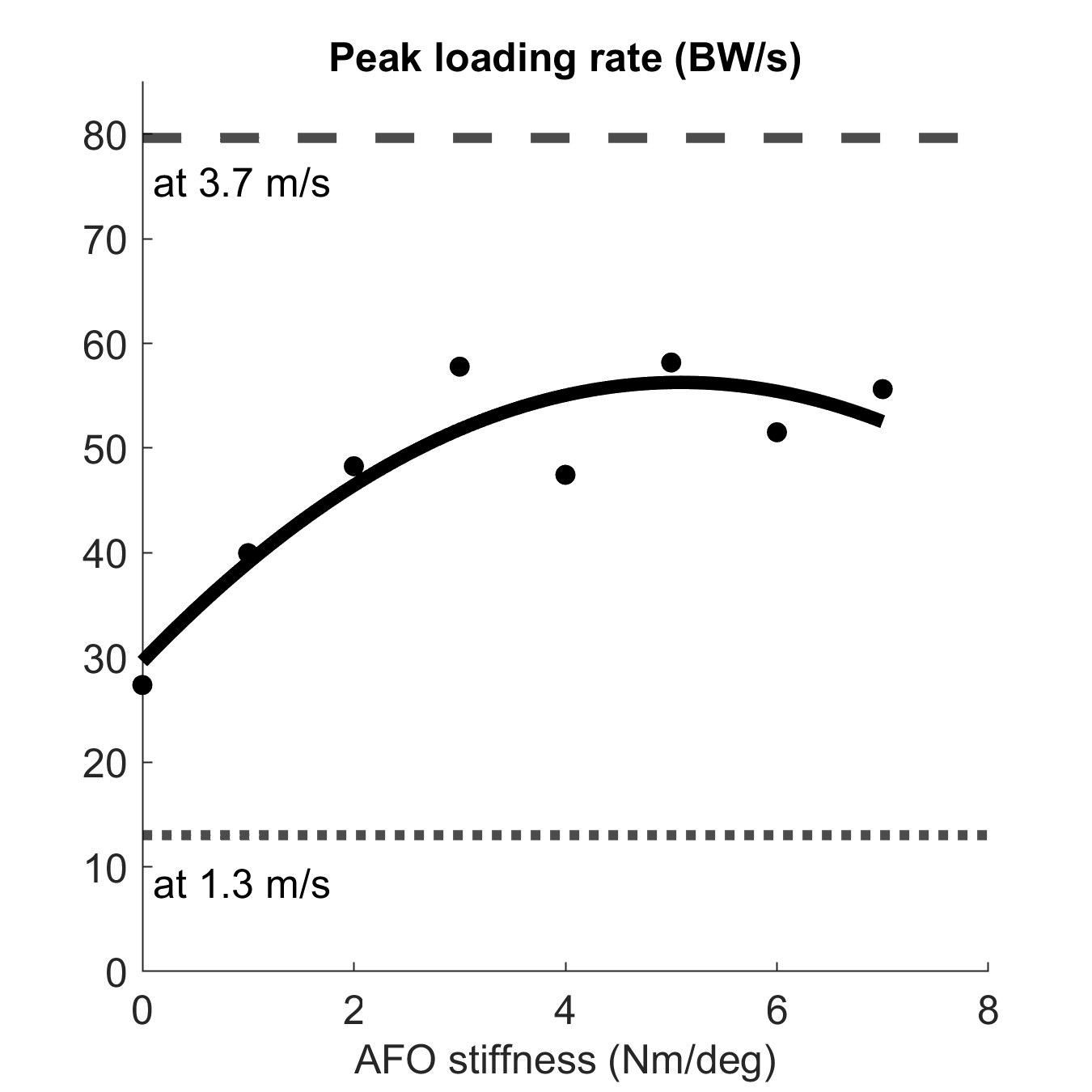

Supplement: Supplementary file 3 [file Image1.TIFF]

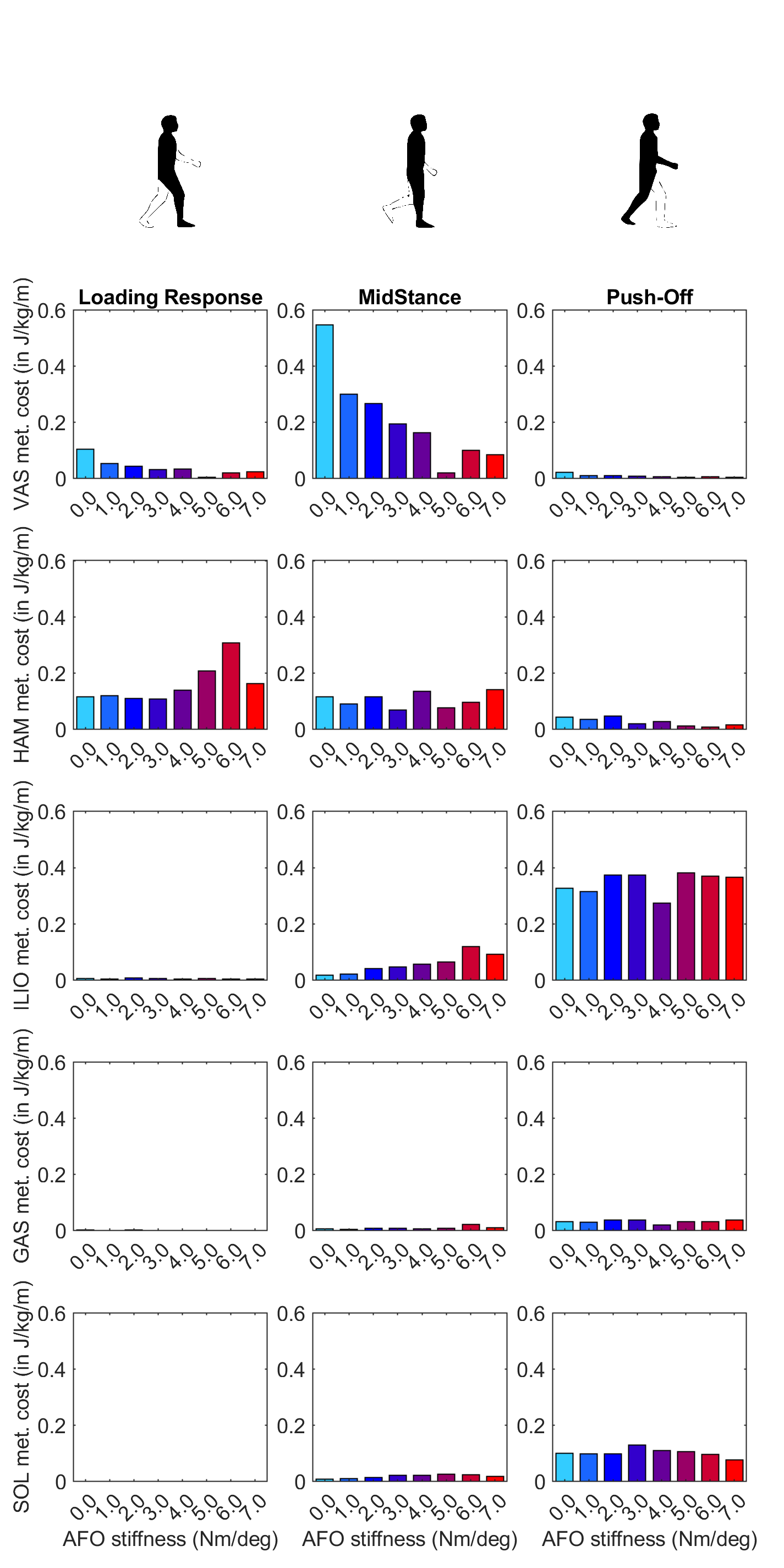

Supplement: Supplementary file 8 [file Image2.TIFF]
