# Supplementary material for: Minimization of metabolic cost of transport predicts changes in gait mechanics over a range of ankle-foot orthosis stiffnesses in individuals with bilateral plantar flexor weakness
Source: Front Bioeng Biotechnol. 2024 May 23;12:1369507. doi: 10.3389/fbioe.2024.1369507 (PMC11153850; doi:10.3389/fbioe.2024.1369507)
Supplement: Supplementary file 2 [file Table5.pdf]

S5 Table. Peak values of relevant joint kinematics, kinetics and powers during the stance phase of gait from the predictive gait simulations. AFO stiffness was varied from 1 − 7 Nm/deg. Negative and positive directions are defined the same as in Fig 2. Negative value means power absorption by ankle/AFO powers, internal dorsiflexion moment by ankle/AFO moments, plantarflexion angle by ankle angles, knee extension by knee angles, and knee flexion moment by knee moments.

**Stiffness** (Nm/deg)	**1**	**2**	**3**	**4**	**5**	**6**	**7**
**peak total ankle pow.** (W/kg)	2.364	1.853	1.796	1.503	1.546	1.385	1.384
**peak AFO pow.** (W/kg)	0.465	0.392	0.466	0.518	0.574	0.562	0.594
**peak biol ankle pow.** (W/kg)	1.917	1.496	1.840	1.359	1.580	1.248	1.124
**peak total ankle mom.** (Nm/kg)	0.979	1.097	1.106	1.150	1.169	1.220	1.177
**peak AFO mom.** (Nm/kg)	0.200	0.316	0.486	0.563	0.612	0.618	0.745
**peak biol ankle mom.** (Nm/kg)	0.791	0.787	0.677	0.645	0.606	0.639	0.516
**peak ankle angle** (deg)	18.463	15.028	15.338	13.606	12.085	10.492	10.768
**peak knee angle** (deg)	1.237	-0.088	0.474	-1.983	-0.235	-2.179	-2.739
**peak knee moment** (Nm/kg)	-0.171	-0.232	-0.181	-0.207	-0.173	-0.186	-0.164
