# Supplementary material for: Minimization of metabolic cost of transport predicts changes in gait mechanics over a range of ankle-foot orthosis stiffnesses in individuals with bilateral plantar flexor weakness
Source: Front Bioeng Biotechnol. 2024 May 23;12:1369507. doi: 10.3389/fbioe.2024.1369507 (PMC11153850; doi:10.3389/fbioe.2024.1369507)
Supplement: Supplementary file 4 [file Table4.pdf]

S4 Table. Metabolic cost of transport of each muscle group calculated with integration for each gait phase. AFO stiffness was varied from 0 − 7 Nm/deg.

Stiffness (Nm/deg)	0	1	2	3	4	5	6	7
**Vasti metabolic cost (J/kg/m)**								
Loading Response	0.105	0.054	0.044	0.032	0.035	0.005	0.021	0.024
MidStance	0.547	0.301	0.268	0.195	0.163	0.020	0.100	0.085
Push-Off	0.023	0.010	0.010	0.009	0.006	0.004	0.006	0.005
Swing	0.021	0.023	0.020	0.021	0.021	0.019	0.021	0.020
**Hamstrings metabolic cost (J/kg/m)**								
Loading Response	0.117	0.121	0.111	0.109	0.141	0.208	0.308	0.164
MidStance	0.117	0.090	0.117	0.070	0.135	0.078	0.097	0.141
Push-Off	0.043	0.035	0.047	0.021	0.029	0.014	0.008	0.017
Swing	0.008	0.007	0.011	0.017	0.023	0.026	0.010	0.016
**Iliopsoas metabolic cost (J/kg/m)**								
Loading Response	0.008	0.006	0.009	0.007	0.005	0.006	0.004	0.004
MidStance	0.018	0.022	0.042	0.047	0.058	0.065	0.121	0.092
Push-Off	0.327	0.315	0.373	0.374	0.274	0.382	0.370	0.367
Swing	0.194	0.231	0.218	0.213	0.294	0.217	0.201	0.277
**Gastrocnemius metabolic cost (J/kg/m)**								
Loading Response	0.002	0.000	0.002	0.000	0.000	0.001	0.001	0.001
MidStance	0.006	0.004	0.009	0.008	0.008	0.009	0.022	0.010
Push-Off	0.032	0.031	0.039	0.038	0.020	0.031	0.032	0.038
Swing	0.004	0.005	0.007	0.008	0.006	0.009	0.007	0.009
**Soleus metabolic cost (J/kg/m)**								
Loading Response	0.001	0.001	0.001	0.001	0.001	0.001	0.001	0.001
MidStance	0.008	0.010	0.015	0.022	0.023	0.026	0.024	0.018
Push-Off	0.102	0.099	0.098	0.130	0.110	0.106	0.097	0.077
Swing	0.007	0.009	0.008	0.008	0.010	0.007	0.008	0.007
**Gluteus maximus metabolic cost (J/kg/m)**								
Loading Response	0.220	0.172	0.180	0.223	0.209	0.216	0.193	0.267
MidStance	0.278	0.231	0.236	0.182	0.196	0.151	0.151	0.157
Push-Off	0.024	0.015	0.030	0.021	0.013	0.019	0.014	0.066
Swing	0.025	0.027	0.026	0.029	0.026	0.026	0.033	0.037
**Rectus femoris metabolic cost (J/kg/m)**								
Loading Response	0.001	0.002	0.002	0.002	0.002	0.002	0.002	0.002
MidStance	0.005	0.004	0.003	0.002	0.003	0.002	0.003	0.003
Push-Off	0.006	0.002	0.000	0.000	0.000	0.001	0.000	0.001
Swing	0.010	0.009	0.008	0.009	0.008	0.010	0.009	0.012
**Biceps femoris short head metabolic cost (J/kg/m)**								
Loading Response	0.003	0.003	0.003	0.002	0.002	0.002	0.002	0.002
MidStance	0.004	0.005	0.005	0.006	0.006	0.006	0.007	0.007
Push-Off	0.005	0.004	0.008	0.004	0.003	0.003	0.025	0.003
Swing	0.007	0.007	0.009	0.007	0.008	0.007	0.046	0.007
**Tibialis anterior metabolic cost (J/kg/m)**								

**Loading Response**	0.017	0.063	0.010	0.052	0.024	0.063	0.031	0.079
**MidStance**	0.020	0.122	0.008	0.121	0.101	0.100	0.079	0.146
**Push-Off**	0.010	0.004	0.002	0.003	0.001	0.003	0.000	0.002
**Swing**	0.182	0.141	0.114	0.116	0.152	0.074	0.079	0.103
