# Supplementary material for: Minimization of metabolic cost of transport predicts changes in gait mechanics over a range of ankle-foot orthosis stiffnesses in individuals with bilateral plantar flexor weakness
Source: Front Bioeng Biotechnol. 2024 May 23;12:1369507. doi: 10.3389/fbioe.2024.1369507 (PMC11153850; doi:10.3389/fbioe.2024.1369507)
Supplement: Supplementary file 5 [file Table1.pdf]

S1 Table. Slope and goodness of fit (Rsq) of line to relevant joint kinematics, kinetics and powers during the stance phase of gait. AFO stiffness was varied from 1 − 7 Nm/deg. From left to right: slope/Rsq of the line fitted to the forward simulation result, mean of the slope/Rsq of the lines fitted to the bilaterally affected patients' results, standard deviation (SD) of the slope/Rsq of the lines fitted to the bilaterally affected patients' result, difference between the simulation slopes and the mean of the experimental slopes divided by the standard deviation of the experimental slopes. Negative and positive directions are defined the same as in Fig 2. Negative slope means change into absorption direction by ankle/AFO powers, change into internal dorsiflexion moment direction by ankle/AFO moments, change into plantarflexion angle direction by ankle angles, change into knee extension direction by knee angles, and change into knee flexion moment direction by knee moments.

		**Sim.**	**Exp.**	**Exp. SD**	**Diff. (in SD)**
**peak total ankle pow.**	**slope** (W/kg per 1Nm/deg)	-0.147	-0.103	0.070	**0.631**
	**Rsq**	0.828	0.611	0.285	
**peak AFO pow.**	**slope** (W/kg per 1Nm/deg)	0.030	-0.007	0.058	**0.633**
	**Rsq**	0.782	0.404	0.349	
**peak biol. ankle pow.**	**slope** (W/kg per 1Nm/deg)	-0.112	-0.091	0.074	**0.289**
	**Rsq**	0.675	0.517	0.366	
**peak total ankle mom.**	**slope** (Nm/kg per 1Nm/deg)	0.032	0.000	0.027	**1.180**
	**Rsq**	0.794	0.317	0.258	
**peak AFO mom.**	**slope** (Nm/kg per 1Nm/deg)	0.085	0.043	0.036	**1.152**
	**Rsq**	0.932	0.663	0.306	
**peak biol. ankle mom.**	**slope** (Nm/kg per 1Nm/deg)	-0.043	-0.029	0.028	**0.469**
	**Rsq**	0.881	0.353	0.351	
**peak ankle angle**	**slope** (deg per 1Nm/deg)	-1.265	-1.124	0.765	**0.184**
	**Rsq**	0.921	0.712	0.264	
**peak knee angle**	**slope** (deg per 1Nm/deg)	-0.601	-0.279	0.670	**0.480**
	**Rsq**	0.740	0.386	0.326	
**peak knee mom.**	**slope** (Nm/kg per 1Nm/deg)	0.004	-0.013	0.025	**0.666**
	**Rsq**	0.153	0.404	0.305	
