# Supplementary material for: Minimization of metabolic cost of transport predicts changes in gait mechanics over a range of ankle-foot orthosis stiffnesses in individuals with bilateral plantar flexor weakness
Source: Front Bioeng Biotechnol. 2024 May 23;12:1369507. doi: 10.3389/fbioe.2024.1369507 (PMC11153850; doi:10.3389/fbioe.2024.1369507)
Supplement: Supplementary file 6 [file Table2.pdf]

S2 Table. Mean metabolic cost for the whole model and for each muscle group of the model. Data taken during a whole gait cycle as AFO stiffness was varied from 0 − 7 Nm/deg.

**Stiffness (Nm/deg)**	**0**	**1**	**2**	**3**	**4**	**5**	**6**	**7**
**Total metabolic cost (J/kg/m)**	4.324	3.975	4.021	3.867	3.861	3.776	3.834	4.118
**Vasti metabolic cost (J/kg/m)**	0.457	0.264	0.248	0.175	0.152	0.035	0.100	0.092
**Hamstrings metabolic cost (J/kg/m)**	0.189	0.175	0.205	0.152	0.225	0.230	0.297	0.234
**Iliopsoas metabolic cost (J/kg/m)**	0.370	0.397	0.462	0.446	0.421	0.486	0.475	0.507
**Gastrocnemius metabolic cost (J/kg/m)**	0.030	0.028	0.041	0.037	0.023	0.036	0.041	0.039
**Soleus metabolic cost (J/kg/m)**	0.080	0.082	0.088	0.109	0.097	0.101	0.087	0.070
**Gluteus maximus metabolic cost (J/kg/m)**	0.357	0.299	0.331	0.315	0.301	0.289	0.269	0.368
**Rectus femoris metabolic cost (J/kg/m)**	0.015	0.012	0.009	0.009	0.009	0.010	0.009	0.012
**Biceps femoris short head metabolic cost (J/kg/m)**	0.013	0.013	0.018	0.013	0.013	0.014	0.052	0.013
**Tibialis anterior metabolic cost (J/kg/m)**	0.157	0.226	0.095	0.201	0.191	0.174	0.128	0.228
