# Supplementary material for: Minimization of metabolic cost of transport predicts changes in gait mechanics over a range of ankle-foot orthosis stiffnesses in individuals with bilateral plantar flexor weakness
Source: Front Bioeng Biotechnol. 2024 May 23;12:1369507. doi: 10.3389/fbioe.2024.1369507 (PMC11153850; doi:10.3389/fbioe.2024.1369507)
Supplement: Supplementary file 7 [file Table3.pdf]

S3 Table. Positive and negative mechanical joint work calculated with integration for each gait phase. AFO stiffness was varied from 0 − 7 Nm/deg.

Stiffness (Nm/deg)	0	1	2	3	4	5	6	7
**Ankle joint work (J/kg)**								
**Loading Response**								
**Positive**	0.000	0.002	0.000	0.000	0.000	0.000	0.000	0.006
**Negative**	-0.046	-0.011	-0.034	-0.012	-0.020	-0.015	-0.016	-0.003
**MidStance**								
**Positive**	0.021	0.000	0.005	0.006	0.009	0.006	0.002	0.006
**Negative**	-0.063	-0.040	-0.050	-0.027	-0.026	-0.023	-0.026	-0.006
**Push-Off**								
**Positive**	0.116	0.123	0.123	0.154	0.129	0.121	0.113	0.093
**Negative**	-0.010	-0.004	-0.002	0.000	0.000	0.000	0.000	0.000
**Swing**								
**Positive**	0.003	0.003	0.003	0.002	0.002	0.003	0.002	0.002
**Negative**	-0.001	-0.004	-0.004	-0.003	-0.007	-0.005	-0.006	-0.005
**AFO work (J/kg)**								
**Loading Response**								
**Positive**	0.000	0.000	0.000	0.000	0.000	0.002	0.000	0.000
**Negative**	0.000	-0.006	-0.009	-0.008	-0.007	-0.004	-0.002	-0.008
**MidStance**								
**Positive**	0.000	0.000	0.004	0.005	0.009	0.007	0.002	0.008
**Negative**	0.000	-0.024	-0.030	-0.049	-0.051	-0.051	-0.044	-0.049
**Push-Off**								
**Positive**	0.000	0.027	0.035	0.051	0.048	0.047	0.043	0.048
**Negative**	0.000	-0.001	-0.002	-0.004	-0.003	-0.003	-0.005	-0.005
**Swing**								
**Positive**	0.000	0.001	0.004	0.003	0.004	0.003	0.005	0.004
**Negative**	0.000	-0.001	-0.002	0.000	-0.001	-0.003	0.000	0.000
**Knee joint work (J/kg)**								
**Loading Response**								
**Positive**	0.029	0.053	0.036	0.046	0.050	0.041	0.039	0.038
**Negative**	-0.123	-0.004	-0.002	0.000	0.000	-0.008	-0.001	0.000
**MidStance**								
**Positive**	0.168	0.047	0.056	0.019	0.010	0.004	0.006	0.004
**Negative**	-0.009	-0.010	-0.023	-0.009	-0.024	-0.057	-0.039	-0.048
**Push-Off**								
**Positive**	0.007	0.013	0.020	0.012	0.014	0.011	0.013	0.011
**Negative**	-0.066	-0.058	-0.055	-0.019	-0.023	-0.026	-0.025	-0.049
**Swing**								
**Positive**	0.004	0.007	0.000	0.009	0.012	0.007	0.006	0.007
**Negative**	-0.116	-0.145	-0.151	-0.150	-0.179	-0.172	-0.148	-0.174

Hip joint work (J/kg)								
**Loading Response**								
**Positive**	0.045	0.053	0.047	0.044	0.051	0.075	0.045	0.049
**Negative**	-0.063	-0.092	-0.058	-0.060	-0.036	-0.019	-0.031	-0.014
**MidStance**								
**Positive**	0.213	0.137	0.136	0.129	0.150	0.173	0.195	0.180
**Negative**	-0.030	-0.035	-0.046	-0.080	-0.087	-0.093	-0.106	-0.101
**Push-Off**								
**Positive**	0.147	0.151	0.168	0.135	0.103	0.149	0.149	0.148
**Negative**	-0.111	-0.081	-0.065	-0.076	-0.053	-0.058	-0.019	-0.030
**Swing**								
**Positive**	0.058	0.079	0.065	0.103	0.126	0.125	0.098	0.112
**Negative**	-0.014	-0.009	-0.009	-0.023	-0.015	-0.020	-0.020	-0.011
