# Supplementary material for: Minimization of metabolic cost of transport predicts changes in gait mechanics over a range of ankle-foot orthosis stiffnesses in individuals with bilateral plantar flexor weakness
Source: Front Bioeng Biotechnol. 2024 May 23;12:1369507. doi: 10.3389/fbioe.2024.1369507 (PMC11153850; doi:10.3389/fbioe.2024.1369507)
Supplement: Supplementary file 9 [file DataSheet1.docx]

Supplementary Figure captions

**Supplementary Figure 1.** Peak loading rate (GRF derivative) in loading response across 0-7 Nm/deg stiffnesses (BW: body weight). The found peak loading rate values are more than twice as high as the loading rate on healthy subjects during walking at 1.3 m/s [50], but lower than the loading rate on healthy subjects during running at 3.7 m/s [59].

**Supplementary Figure 2.** Vasti, hamstrings, iliopsoas, gastrocnemius, and soleus metabolic cost during loading response, midstance, and push-off in simulation. AFO stiffness was varied from 0-7 Nm/deg. Knee joint work was portioned into positive and negative work and these were totaled and reported for each phase of gait. Abbreviations: VAS: vasti muscle group, HAM: hamstrings muscle group, ILIO: iliopsoas muscle group, GAS: gastrocnemius muscle, SOL: soleus muscle. The metabolic cost of transport values of each muscle group calculated with integration for each gait phase can be found in Table S4.

**Supplementary Figure 3.** Biological hip, knee, and ankle joint work, and AFO work during loading response, midstance, and push-off in simulation. AFO stiffness was varied from 0-7 Nm/deg. Biological hip, knee, and ankle joint work, and AFO work was portioned into positive and negative work and these were totaled and reported for each phase of gait. The values of the positive and negative mechanical joint work calculated with integration for each gait phase can be found in Table S3.
